# Functional Reconstruction of Severely Burned Hand With Osseous Blood Flow Deficiency With Immediate Surgery Using an Abdominal Bipediceled Flap: A Case Report

**Published:** 2018-02-23

**Authors:** Masakatsu Hihara, Takashi Matsushima, Yoshihito Tanaka, Yutaka Ogawa, Natsuko Kakudo, Kenji Kusumoto

**Affiliations:** ^a^Department of Plastic and Reconstructive Surgery, Kansai Medical University, Hirakata, Japan; ^b^Division of Plastic and Reconstructive Surgery, Moriyama Municipal Hospital, Moriyama, Japan

**Keywords:** third-degree burn, hand burn, immediate operation, osseous blood flow deficiency, finger reconstruction

## Abstract

**Objective:** When hands suffer burns, the tendons and digital bones are rarely injured because of the quick withdrawal reflex away from the heat source. Hence, a consensus of opinion regarding the treatment of severe hand burns with osseous blood flow deficiency has not been reached among clinicians. **Methods:** The patient was a 28-year-old woman whose fingers were accidentally soaked in heated cooking oil (160°C-170°C) for approximately 1 minute. The result was fourth-degree (extending to the tendon) digital burns of the distal end of the proximal interphalangeal joint with blood flow deficiency to the skin, tendon, and bone. **Results:** We performed immediate reconstructive surgery using an abdominal bipediceled flap. Two weeks later, after the flap was separated, all fingers showed complete range of motion, restoration of the metacarpophalangeal joint, and a high range (0°-75°) of proximal interphalangeal joint mobility with an acceptable digital length. **Conclusions:** Our experience shows that immediate surgery is highly preferable for deep burns of the hand to avoid delayed intractable complications and to achieve better and faster results.

A large number of studies have been conducted on the management of deep burns, and it has been pointed out that immediate operation is useful in cases of severe burns. In particular, in cases of severely burned hands, it seems that surgery leads to a better functional result than conservative treatment.^1^^,^^2^ However, a consensus of opinion among clinicians has not been reached. We report a case of severe hand burn with osseous blood flow deficiency that was successfully treated by an immediate operation with an abdominal bipediceled flap.

## CASE REPORT

**Figure 1:**
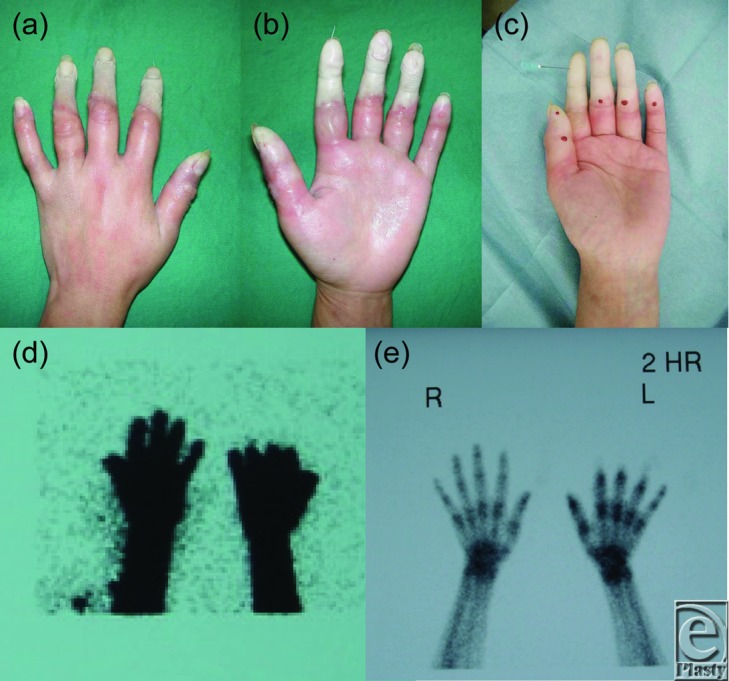
Dorsal (a) and palmar views (b) of the left hand on admission. The burned skin was already accompanied by tissue degeneration. A prick test was performed to the middle phalanx with a 23-G needle, but no bleeding was observed beyond the PIP joint; even exudation was not noted (c). In an early blood flow scintigraphy image using Tc 99m HMDP, blood flow absence was shown (d). In a bone metabolism image, bones beyond the PIP joints were not circulatory supplied (e). PIP indicates proximal interphalangeal.

A 28-year-old woman sustained a serious burn to her left hand. While cooking, she experienced an atypical absence seizure of intractable epilepsy of temporal lobe lesion origin. Her left fingers were accidentally soaked in tempura oil (160°C-170°C) for around 1 minute. On admission, deeper (extending to the tendons, ie, fourth-degree) digital burns of the distal ends of the proximal interphalangeal (PIP) joints were shown (Figs 1*a* and 1*b*). Blood flow to the fingertips was completely absent, and the skin was already showing signs of tissue degeneration. A pin prick test was performed to the middle phalanx bone with a 23-G needle, but no bleeding, nor even exudation, was observed beyond the PIP joint (Fig 1*c*). Since tendon and bone blood flow deficiency was strongly suspected, bone blood flow scintigraphy using Tc 99m HMDP (technetium-99m hydroxymethylene diphosphonate) was planned to scan for blood flow in the deep part. Scintigraphy showed blood flow absence in the distal phalanx of the index to ring fingers, and a bone metabolism image showed osteonecrosis pattern of these parts (Figs 1*d* and 1*e*). On the basis of these results, we explained to the patient and her family that digital amputation was unavoidable, but their strong desire to preserve the length of the digits led us to perform surgery.

**Figure 2:**
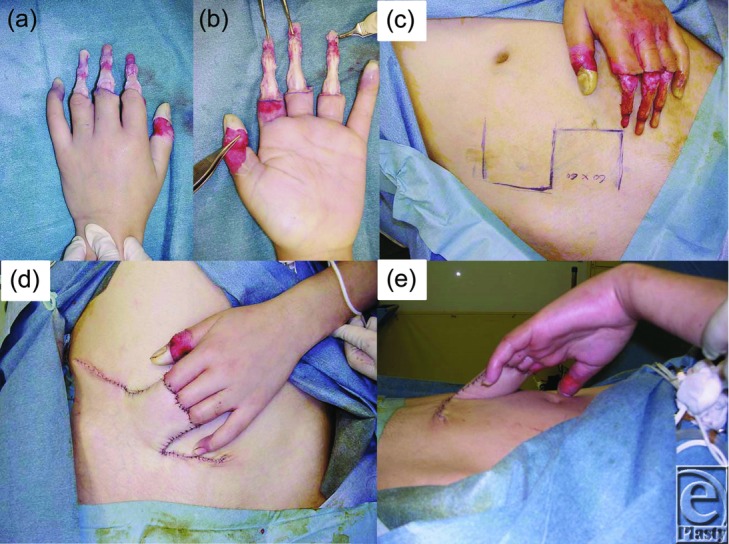
Intraoperative dorsal (a) and palmar views (b) just after debridement of tissues except the bones and tendons. A 6 × 6-cm abdominal flap was harvested (c). Frontal (d) and lateral views (e) of the harvested flap covering the bones and tendons.

**Figure 3:**
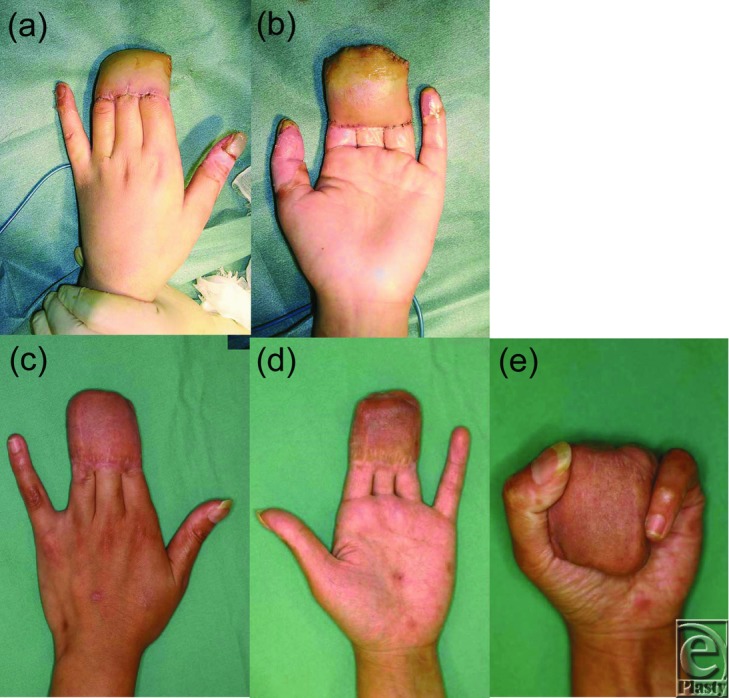
Dorsal (a) and palmar views (b) of the left hand 2 weeks postoperatively. Dorsal (c) and palmar views (d) of the left hand 9 months postoperatively. Active flexion after 9 months of training (e).

**Figure 4:**
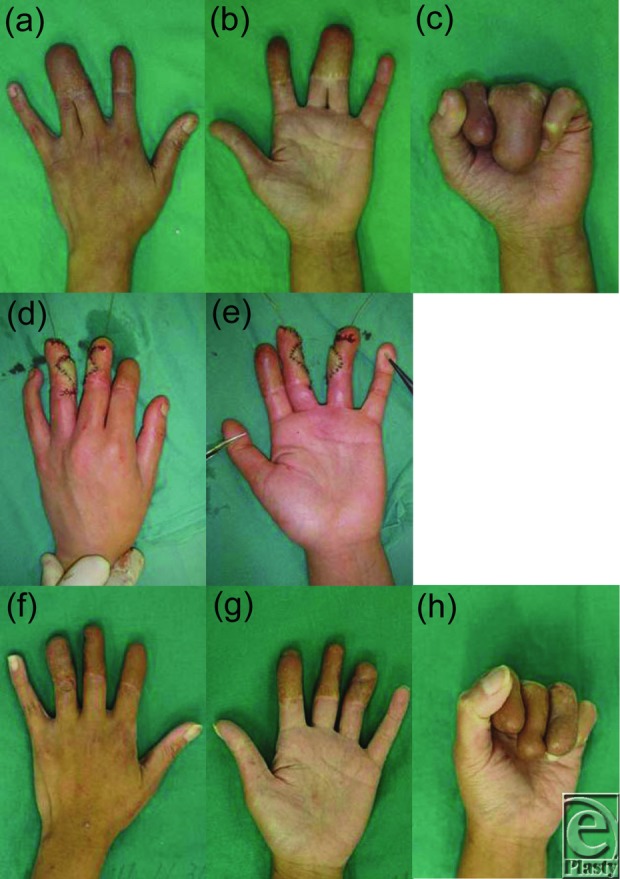
Dorsal (a) and palmar views (b) of the left hand 1 year and 5 months postoperatively. Active flexion after 1 year and 5 months of training (c). Intraoperative dorsal (d) and palmar views (e) of middle-ring finger separation with grafting. Dorsal (f) and palmar views (g) of the left hand 2 years postoperatively. Active flexion after 2 years of training (h). Functional restoration of digits with an acceptable length. The range of motion of the metacarpophalangeal joint was fully restored and that of the proximal interphalangeal joint was 0° to 75°.

The procedure was performed under general anesthesia 24 hours after injury. The eschar was already showing marked alteration. At first, all nonviable eschar including the neurovascular bundle was removed surgically. Thereafter, the bones and tendons were exposed without the surrounding soft tissue (Figs 2*a* and 2*b*). Blood flow was lacking in the remaining tissue. A conventional abdominal flap of 6 × 6 cm including superficial fascia was raised in the left lower quadrant to cover the naked tendons and bones of the fingers in a block (Figs 2*c*–2*e*). The pedicle of the flap was cut and detached from the abdomen 2 weeks later, following an eventual course to produce provisional syndactyly between the index to ring fingers (Figs 3*a* and 3*b*). In a state of provisional syndactyly, range of motion (ROM) exercise of the metacarpophalangeal (MCP) and PIP joints was performed for 9 months (Figs 3*c*–3*e*). Since resurfacing of the burned tendons and bones with the flap was successfully performed, separation of the syndactyly was performed between the index and middle fingers (Figs 4*a*–4*c*). Separation of the middle and ring fingers was performed with skin grafting 1 year and 5 months later (Figs 4*d* and 4*e*). After rehabilitation for 2 years, all fingers showed complete ROM, restoration of the MCP joint, and a high range (0°-75°) of PIP joint mobility with an acceptable digital length (Figs 4*f*–4*h*).

## DISCUSSION

In the case of burned hands, the tendons and digital bones are rarely injured because of the quick escape response away from the heat source. In our case, the patient could not remove her fingers from the hot oil due to unconsciousness. Although conservative treatment may dominate in judging the debridement of necrotic tissue, the waiting period may be longer than the window of opportunity to restore joint mobility, resulting in boutonniere deformity by palmer displacement of the lateral band. Furthermore, as indirect heat damage, peripheral circulatory disturbance causes compartment syndrome; fingertip necrosis is shown occasionally. Immediate surgery should be performed to avoid postburn infection and facilitate easy management to resurface the wound.

Our basic strategy was that the remnant tissue should be relieved by immediate excision of the necrotic tissue. There was no doubt that ischemic changes existed in the digits on her admission, as shown via scintigraphy in the low blood flow image and the osteonecrotic image. Although specific thermal damage was still unknown, we planned a procedure used for degloving injuries to reconstruct the naked fingers. The severity of the case seemed much worse than simple degloving injury because of the osseous ischemia. The risk of osteonecrosis remained, since the bones and tendons were devoid of any blood supply from the digital artery in addition to having suffered direct heat damage. However, it must be more advantageous for osseous survival than merely nonvascularized bone transplantation to maintain the digital length, because the middle phalanx may obtain some blood flow through the periosteum from the normal proximal phalanx; besides, in later years, a heat-treated bone (eg, Pasteur disposal bones) can be used for reconstruction as a disengagement bone, where the graft achieves a high survival rate regardless of thermophile treatment.^3^ This suggests that direct heat damage influences the viability of bone tissue less than other tissues.

On the contrary, this may be true of the tendon, which metabolically shows a low activity. When blood flow to the periosteum and mesotendon is reopened by covering with sufficient viable tissue in the early period, the bones and tendons are protected from damage and resorption. Flap covering resumes blood flow to the vincula through the mesotendon and the periosteum, which ensures the viability of tissue.^4^ As a result, an acceptable length of fingers and functional PIP joints could be obtained by the abdominal flap. Although the mechanism of blood flow resumption was not clarified in this case, blood flow to the tendons was resumed and normal tendon function could be restored.

As other choices for reconstruction, the free vascularized thin flap (such as rectus abdominis perforator flap, groin flap, anterolateral thigh flap, or latissimus dorsi muscle flap) was available after eschar removal.^5^^,^^6^ Since tendon and bone exposure in circumferential hand burns makes early rehabilitation difficult, a free flap may be indicated, but it is too difficult to resurface multiple fingers simultaneously. We do emphasize that finger function might not be restored if debridement is performed after long-term conservative treatment without immediate surgery. So, when successful covering is achieved in the case, the salvaging of necrotizing bones and tendons may be possible.
